# Does type 2 diabetes influence the risk of oesophageal adenocarcinoma?

**DOI:** 10.1038/sj.bjc.6604908

**Published:** 2009-02-03

**Authors:** R E Neale, J D Doecke, N Pandeya, S Sadhegi, A C Green, P M Webb, D C Whiteman

**Affiliations:** 1 Cancer and Population Studies Group, Queensland Institute of Medical Research, Queensland, Australia; 2 School of Population Health, University of Queensland, Brisbane, Queensland, Australia

**Keywords:** oesophageal neoplasms, diabetes mellitus, case–control study

## Abstract

Since hyperinsulinaemia may promote obesity-linked cancers, we compared type 2 diabetes prevalence among oesophageal adenocarcinoma (OAC) patients and population controls. Diabetes increased the risk of OAC (adjusted odds ratio 1.59, 95% confidence interval (CI) 1.04–2.43), although the risk was attenuated after further adjusting for body mass index (1.32, 95% CI 0.85–2.05).

Adenocarcinomas of the oesophagus (OAC) and gastro-oesophageal junction (GOJAC) have been rising rapidly in incidence in many countries (Blot *et al*, 1991; Wayman *et al*, 2001; Pohl and Welch, 2005), widely attributed to the steadily rising prevalence of obesity (Silventoinen *et al*, 2004; Reeves *et al*, 2007; Whiteman *et al*, 2007; Corley *et al*, 2008).

One proposed mechanism to explain the association between obesity and OAC is that obesity increases the risk of OAC through the pathway of gastro-oesophageal acid reflux. However, we and others have shown that obesity is strongly associated with OAC (Corley *et al*, 2008) even among people with no history of reflux (Chow *et al*, 1998; Whiteman *et al*, 2007), arguing against increased exposure to acid as being the sole mechanism for the observed phenomenon. A second postulated mechanism is that the metabolic changes accompanying adiposity establish a hormonal *milieu* that promotes the development of nascent tumours (Calle and Kaaks, 2004).

A candidate hormone central to the ‘metabolic hormonal hypothesis’ is insulin. Insulin activates the insulin receptor, triggering intracellular signalling cascades with mitogenic and antiapoptotic effects (Calle and Kaaks, 2004). In addition, its positive feedback influences levels of leptin, a mitogenic adipocytokine that has been implicated in cancers of the colon, breast and prostate (Tessitore *et al*, 2000; Stattin *et al*, 2003). If hyperinsulinaemia is positively associated with the risk of OAC, then an association with type 2 diabetes (a proxy for pre-existing hyperinsulinaemia) should be observed, independently of other obesity-related factors.

Although some studies investigated oesophageal cancer in relation to diabetes, only three distinguished adenocarcinomas from squamous cell carcinomas (Cheng *et al*, 2000; Reavis *et al*, 2004; Rubenstein *et al*, 2005). For each study, however, the prevalence of diabetes among controls was markedly different from prevailing population estimates. We therefore used data from a large population-based case–control study to explore the hypothesis and address some gaps in the evidence.

## Methods

The study was approved by the human research ethics committees of the Queensland Institute of Medical Research and participating hospitals and we obtained written informed consent from all participants. Details of recruitment and data collection have been reported earlier (Whiteman *et al*, 2007). Briefly, patients (cases) with a primary invasive OAC or oesophageal squamous cell carcinoma (OSCC) or diagnosed between 1 July 2001 and 30 June 2005 were recruited through major treatment centres and state-based cancer registries throughout mainland Australia. In all, 1102 patients returned questionnaires (75% of those meeting the inclusion criteria and invited to take part: 367 OAC, 426 GOJAC and 309 OSCC).

Potential controls were randomly selected from the Australian electoral roll (enrolment is compulsory), matched to the distribution of the case-series by age, sex and state of residence. Completed questionnaires were returned by 1580 controls (52% of all controls meeting the inclusion criteria).

Data were collected through self-completed questionnaires about education, occupation, smoking, medication use, maximum weight and height and weight 1 year ago. We calculated BMI by dividing weight in kilograms by the square of height in metres. Participants were asked about the frequency of symptoms of gastro-oesophageal reflux. For analysis, we defined ‘frequent symptoms’ as those occurring at least weekly.

Diabetes was self-reported using the questions ‘Have you ever had diabetes treated with insulin injections?’ and ‘Have you ever had diabetes treated with tablets and/or diet?’ A positive response to either of these questions prompted a question about the age of diagnosis. We classified those who reported diabetes treated only with tablets/diet as having type 2 diabetes. Participants who had insulin-treated diabetes were considered to have type 2 diabetes if they were diagnosed after the age of 25 years.

Details of the histological type and anatomical site of each tumour were abstracted from diagnostic pathology reports. Tumours were categorised according to the WHO classification as ‘oesophageal’ or of the ‘gastro-oesophageal junction’ (Spechler *et al*, 2000). We calculated the odds ratios (ORs) and 95% confidence intervals (95% CIs) associated with diabetes using multivariable logistic regression analysis in SAS version 9.1 (SAS Institute Inc., Cary, NC). We first fitted models that contained terms for diabetes, age and sex (Model 1). We then estimated risks associated with diabetes adjusted for these variables and education, smoking, frequency of aspirin use and frequency of gastro-oesophageal reflux symptoms (Model 2). Finally, we fitted models comprising the preceding variables as well as BMI (Model 3).

## Results

The demographic and clinical characteristics of cases and controls have been reported earlier (Sadeghi *et al*, 2008). Diabetes was self-reported by 7% of controls, compared with 14, 12 and 4% of cases who had OAC, GOJAC and OSCC, respectively.

In simple models adjusted only for age and sex, we found a significant increase in the risk of OAC associated with diabetes (Model 1: OR 1.78, 95% CI 1.22–2.62) that was somewhat attenuated by adjustment for additional factors (Table 1). Adding the term for BMI to the model further attenuated the estimate, with the final OR suggesting a 32% increase in risk of OAC associated with diabetes, although this increase was no longer statistically significant (Model 3: OR 1.32, 95% CI 0.85–2.05). Similar patterns were observed for GOJAC, although the magnitude of association was smaller. There was some suggestion that people whose diabetes had been diagnosed greater than 10 years before their adenocarcinoma had a greater risk than those whose onset of diabetes was more recent (Table 1).

We observed consistently higher risks of OAC among those with diabetes within each category of BMI. People with diabetes who were also obese were at a 3.5-fold higher risk of OAC than those with neither risk factor (OR 3.55, 95% CI 1.87–6.76). Oesophageal adenocarcinoma risks were somewhat lower in those with either obesity or diabetes alone (obese OR 2.67, 95% CI 1.80–3.96; diabetes OR 1.86, 95% CI 0.65–5.31). In contrast to OAC, the odds of OSCC were significantly lower in people with diabetes compared with those without (OR 0.54, 95% CI 0.29–1.0) (Table 1).

## Discussion

Obesity is an established risk factor for OAC, although the precise mechanism involved remains unknown. An emerging hypothesis is that metabolic aberrations accompanying obesity lead to changes in hormones and cytokines, including insulin. We indirectly tested this ‘metabolic hormonal hypothesis’ by comparing the diabetes prevalence among oesophageal cancer patients with population controls, and found that those with adenocarcinoma were more likely than controls to report a history of diabetes. The association was greater for long-standing diabetes than for more recent diagnoses. In contrast, we found the suggestion of an inverse association between diabetes and OSCC.

The risk estimates generated by different multivariable models warrant comment. As excess body fat leads to high levels of insulin (and eventually to diabetes) and as diabetes is a proxy measure for hyperinsulinaemia, the inclusion of terms for both of these factors in the same model may underestimate the real effect of hyperinsulinaemia. One could argue that the effect of hyperinsulinaemia is best estimated by Model 2, which adjusts the association between diabetes and OAC for factors that might explain the confounding effect of BMI (e.g., gastro-oesophageal reflux), but without overadjusting the association with hyperinsulinaemia (Rothman, 2002).

To address potential selection bias in controls, we compared the age-specific prevalence of diabetes in our control group with that reported in the population-based Australian Diabetes Survey (Dunstan *et al*, 2001) and found a very high concordance. Patients may have lost weight before their diagnosis, so we performed all analyses using maximum reported BMI, with negligible difference in the results.

We found only three earlier studies that investigated the association between diabetes and OAC (Cheng *et al*, 2000; Reavis *et al*, 2004; Rubenstein *et al*, 2005). Of these, one found a substantial and significant increase in risk of OAC with diabetes (Reavis *et al*, 2004), one reported an OR of close to one (Rubenstein *et al*, 2005) and the third a highly unstable risk estimate of 7.00 (95% CI 0.85–56.89) (Cheng *et al*, 2000). In all three studies, the prevalence of diabetes among controls was different from population estimates, raising concerns about generalisability.

In summary, our data support the hypothesis that diabetes is associated with an increased risk of OAC and that this is unlikely to be due entirely to other factors associated with a high BMI. Resolution of this question requires a further analysis of existing or new population-based data sets, in which histological subtypes are treated as separate entities.

## Figures and Tables

**Table 1:** Relative risks for adenocarcinomas of the oesophagus (OAC), gastro-oesophageal junction (GOJAC) and squamous cell carcinoma (OSCC) associated with type 2 diabetes; minimally, partially and fully adjusted for confounding factors

**History of type 2 diabetes**	**Control (*N*)**	**Case (*N*)**	**OR (95% CI)^a^**	**OR (95% CI)^b^**	**OR (95% CI)^c^**
*OAC*					
No diabetes	1423	293	1.0 (ref)	1.0 (ref)	1.0 (ref)
Diabetes	103	46	1.78 (1.22–2.62)	1.59 (1.04–2.43)	1.32 (0.85–2.05)
					
No diabetes	1423	293	1.0 (ref)	1.0 (ref)	1.0 (ref)
Diabetes duration <10 years	68	25	1.46 (0.90–2.38)	1.39 (0.82–2.36)	1.14 (0.66–1.97)
Diabetes duration ⩾10 years)	30	18	2.45 (1.32–4.57)	1.96 (0.97–3.97)	1.65 (0.80–3.40)
*P*-trend					0.18
					
*GOJAC*					
No diabetes	1423	338	1.0 (ref)	1.0 (ref)	1.0 (ref)
Diabetes	103	44	1.56 (1.06–2.28)	1.44 (0.96–2.16)	1.23 (0.82–1.87)
					
No diabetes	1423	338	1.0 (ref)	1.0 (ref)	1.0 (ref)
Diabetes (duration <10 years)	68	20	1.06 (0.63–1.79)	0.90 (0.57–1.71)	0.87 (0.50–1.51)
Diabetes (duration ⩾10 years)	30	22	2.76 (1.55–4.91)	2.46 (1.33–4.55)	2.16 (1.16–4.04)
*P*-trend					0.07
					
*OSCC*					
No diabetes	1423	270	1.0 (ref)	1.0 (ref)	1.0 (ref)
Diabetes	103	12	0.54 (0.29–1.0)	0.48 (0.25–0.91)	0.57 (0.30–1.10)
					
No diabetes	1423	270	1.0 (ref)	1.0 (ref)	1.0 (ref)
Diabetes (duration <10 years)	68	7	0.49 (0.22–1.08)	0.42 (0.18–0.97)	0.52 (0.23–1.20)
Diabetes (duration ⩾10 years)	30	5	0.73 (0.28–1.92)	0.62 (0.23–1.67)	0.70 (0.25–1.93)
*P*-trend					0.17

CI=confidence interval; OR=odds ratio.

a OR: Model 1: adjusted for age (in years) and sex.

b OR: Model 2: adjusted for age (in years), education level, cumulative smoking history, frequency of gastro-oesophageal reflux symptoms 10 years before diagnosis and frequency of aspirin use in the last 5 years.

c OR: Model 3: as for Model 2 with an additional term for BMI.

## Appendix

The Australian Cancer Study: Oesophageal Cancer Contributors

Investigators: David C Whiteman MBBS, PhD, Penelope M Webb MA, D Phil, Adele C Green MBBS, PhD, Nicholas K Hayward PhD, Peter G Parsons PhD and David M Purdie PhD.

Clinical collaborators: B Mark Smithers FRACS, David Gotley FRACS PhD, Andrew Clouston FRACP PhD and Ian Brown FRACP.

Project manager: Suzanne Moore RN, MPH.

Research nurses: Suzanne O’Brien RN MPH, Ellen Minehan RN, Deborah Roffe RN, Sue O’Keefe RN, Suzanne Lipshut RN, Gabby Connor RN, Hayley Berry RN, Frances Walker RN, Teresa Barnes RN, Janine Thomas RN, Linda Terry RN MPH, Michael Connard B Sc, Leanne Bowes B Sc, MaryRose Malt RN and Jo White RN.

Database: Karen Harrap BIT and Troy Sadkowski BIT.

Clinical contributors:

Australian Capital Territory: Charles Mosse FRACS, Noel Tait FRACS New South Wales: Chris Bambach FRACS, Andrew Biankan FRACS, Roy Brancatisano FRACS, Max Coleman FRACS, Michael Cox FRACS, Stephen Deane FRACS, Gregory L Falk FRACS, James Gallagher FRACS, Mike Hollands FRACS, Tom Hugh FRACS, David Hunt FRACS, John Jorgensen FRACS, Christopher Martin FRACS, Mark Richardson FRACS, Garrett Smith FRACS, Ross Smith FRACS and David Storey FRACS.

Queensland: John Avramovic FRACS, John CrEse FRACP, Justin D’Arcy FRACS, Stephen Fairley FRACP, John Hansen FRACS, John Masson FRACP, Les Nathanson FRACS, Barry O’Loughlin FRACS, Leigh Rutherford FRACS, Richard Turner FRACS, Morgan Windsor FRACS South Australia: Justin Bessell FRACS, Peter Devitt FRACS, Glyn Jamieson FRACS and David Watson FRACS.

Victoria: Stephen Blamey FRACS, Alex Boussioutas FRACP, Richard Cade FRACS, Gary Crosthwaite FRACS, Ian Faragher FRACS, John Gribbin FRACS, Geoff Hebbard FRACP, George Kiroff FRACS, Bruce Mann FRACS, Bob Millar FRACS, Paul O’Brien FRACS, Robert Thomas FRACS and Simon Wood FRACS.

Western Australia: Steve Archer FRACS, Kingsley Faulkner FRACS and Jeff Hamdorf FRACS.
