# Does type 2 diabetes influence the risk of oesophageal adenocarcinoma?

**DOI:** 10.1038/sj.bjc.6605067

**Published:** 2009-04-28

**Authors:** R E Neale, J D Doecke, N Pandeya, S Sadeghi, A C Green, P M Webb, D C Whiteman

**Correction to:** British Journal of Cancer (2009) **100,** 795–798; doi:10.1038/sj.bjc.6604908

During submission of the above article to the journal, one of the authors’ surnames was spelt incorrectly – S Sadeghi's name was published as ‘S Sadhegi’.

The authors are now happy to correct this mistake.

